# The Effects of the Barbell Hip Thrust on Post-Activation Performance Enhancement of Change of Direction Speed in College-Aged Men and Women

**DOI:** 10.3390/sports8120151

**Published:** 2020-11-24

**Authors:** Ashley J. Orjalo, Samuel J. Callaghan, Robert G. Lockie

**Affiliations:** 1 Department of Kinesiology, California State University, Fullerton, Fullerton, CA 92831, USA; ashley.orjalo@csu.fullerton.edu (A.J.O.); rlockie@fullerton.edu (R.G.L.); 2 Faculty of Sport, Applied Health and Performance Sciences (SAHPS), St Mary’s University, Twickenham TW1 4SX, UK

**Keywords:** agility, college-aged, complex training, hip extension, lower-body strength

## Abstract

This study investigated whether the barbell hip thrust (BHT) enhanced change-of-direction (COD) speed measured by the 505 COD speed test. Forty recreationally trained individuals completed three sessions. Session 1 included one-repetition maximum (1RM) BHT testing to measure absolute and relative strength. Sessions 2 and 3 involved two counter-balanced conditioning activities (CAs): 3 sets × 5 repetitions of the BHT at 85% 1RM and a control condition (CC; 6 min rest). The 505 COD speed test was performed 5 and 2.5 min pre-CA, and 4, 8, 12, and 16 min post-CA in each session. A 2 × 5 repeated-measures ANOVA (*p* < 0.05) calculated performance changes across time post-CA. A 2 × 2 repeated-measures ANOVA analyzed best potentiated performance. Partial correlations controlling for sex calculated relationships between the 1RM BHT and 505 COD speed test percent potentiation. There was a significant main effect for time (*p* < 0.001), but not for condition (*p* = 0.271) or condition × time (*p* = 0.295). There were no significant correlations between 1RM BHT and potentiation. The 85% 1RM BHT did potentiate the 505 4–16 min post-CA but no more than the CC. Nonetheless, a heavy BHT could be programmed prior to COD drills as COD speed could be potentiated and performance improved in men and women.

## 1. Introduction

Agility is an essential quality of many athletes and has been defined as an action that features an initiation of body movement, change-of-direction (COD), or rapid acceleration or deceleration in response to a stimulus [1]. In addition to the cognitive component, the physical component of agility is termed COD speed. COD speed involves factors such as the athlete’s sprint technique, strength, and power [1]. Explosive and decisive COD movements are undertaken within the match-play of many individual and team-based sports and are often central to success. For example, a line break in rugby league, attempting a catch in cricket or a fast break in basketball. Accordingly, different training methods have been used to improve COD speed. This includes sprint training, plyometrics, specific COD drills, the flywheel paradigm, and resistance training [2,3,4,5]. One training method for acute changes in COD speed that has received limited analysis involves post-activation performance enhancement (PAPE), particularly for field- or court-based sports.

PAPE is where a muscle’s contractile history contributes to enhanced power-based actions [6]. Specifically, changes in muscle temperature, muscle/cellular water content, and muscle activation have been suggested to partially explain the effect of increased force production and enhanced power-based actions [7]. This is typically achieved through complex training, which involves a superset combining a strength exercise (or a high force output exercise such as plyometrics) immediately followed by a power-based exercise (e.g., a jump or sprint) [8]. The first exercise in the complex pair is often referred to as a conditioning activity (CA). There has been some analysis of PAPE and COD speed [9,10,11]. Although not a true representation of COD speed, Okuno et al. [9] investigated whether a CA of back squats (1 set × 5 repetitions at 50% one-repetition maximum (1RM), 1 set × 3 repetitions at 70% 1RM, and 5 sets × 1 repetition at 90% 1RM) could enhance repeated-sprint ability test (6 × 30 m sprints with a 180° COD at 15 m) in male handball players. The results suggested that the best and mean times from the six sprints were faster following the CA. Sole et al. [10] also analyzed the use of the back squat as a CA for a 10 meter (m) shuttle run test, which required participants to perform short, maximal sprints interspersed by two 180° change of directions, in collegiate male and female athletes. Although there was a not a significant decrease in shuttle run times 4, 8, and 12 min post-CA, Sole et al. [10] noted that due to individual responses, there was potential application of PAPE for COD speed. Orjalo et al. [11] analyzed whether 3 sets × 5 repetitions of lateral bounds (unweighted and performed with an additional load of 10% body mass) could enhance COD speed as measured by the 505 COD speed test. The results suggested that the overload provided by lateral bounds was not sufficient to potentiate 505 COD speed test time. One of the recommendations from Orjalo et al. [11] was to analyze whether heavy-resistance exercises were more appropriate to induce a PAPE effect on COD speed.

The overload provided by the CA, in addition to the biomechanical specificity between the CA and power-based exercise, is an important factor in determining whether PAPE occurs [6,12]. For example, one reason why Sole et al. [10] may not have found significant changes to shuttle times following the back squat is that the back squat emphasizes vertical force production [13]. Whereby, sprint acceleration, which is a component of COD speed [1], places a greater emphasis on horizontal force [14]. An exercise that may target the muscles responsible for horizontal force production in sprinting is the barbell hip thrust (BHT). The hip extensors (i.e., the gluteals) are the prime movers of the body during the stance phase of sprinting [15]. When compared to a 10RM back squat, the 10RM BHT led to greater gluteus maximus and biceps femoris activation [16]. There is some evidence as to the benefits of using the BHT to potentiate linear sprint performance [17,18]. The BHT performed with 3 sets of 6 repetitions at 85% 1RM enhanced 10- and 15 m sprint times in handball players [17], and 5, 10, and 20 m sprint times in soccer players [18], 4 and 8 min post-CA. Due to the importance of acceleration to COD speed [1], it would be worth investigating whether the 85% 1RM BHT could potentiate COD speed as measured by a task such as the 505 COD speed test.

Training history can also influence whether an individual experiences PAPE [12,18,19,20]. Stronger individuals can potentiate sooner following a strength intervention [20,21], and greater peak force and power production during a resistance exercise could positively influence sprint PAPE [22]. However, the extent to which training history influences COD speed performance among field- and court-based athletes has not been appropriately investigated. There was a relationship between a greater 1RM BHT with larger 15 [17] and 20 m [18] sprint potentiation in handball and soccer players, respectively. In addition to determining whether the BHT can enhance COD speed measured by the 505 COD test, it is important to document whether greater absolute and relative BHT strength can influence the timing and degree of PAPE specific to the 505 COD speed test among field- and court-based athletes.

Therefore, the purpose of this research was to determine whether 3 sets of 5 repetitions at 85% 1RM of the BHT could potentiate COD speed measured by the 505 COD speed test in college-aged men and women. It was hypothesized that the BHT would lead to a faster 505 performance compared to a control condition (CC) of 6 min rest. Lastly, it was hypothesized that there would be significant relationships between absolute and relative BHT load and percent potentiation in the 505 COD speed test [17,18].

## 2. Materials and Methods

### 2.1. Subjects

A convenience sample of 40 subjects (23.28 ± 2.82 years; 1.70 ± 0.09 m; 73.00 ± 12.95 kg) were recruited for this study, including 20 males (23.95 ± 3.24 years; 1.77 ± 0.06 m; body mass: 80.46 ± 12.15 kg) and 20 females (22.60 ± 2.21 years; height: 1.63 ± 0.06 m; body mass: 65.54 ± 8.92 kg). Similar to Orjalo et al. [11], G*Power software (v 3.1.9.3, Universität Kiel, Düsseldorf, Germany) confirmed post hoc that 40 subjects within a 2 × 5 repeated-measures analysis of variance (ANOVA,) indicated data could be interpreted with an effect level of 0.2 and power level of 0.9, when significance was set at 0.05 [23]. Furthermore, 40 subjects for the 2 × 2 repeated-measures ANOVA allowed data to be interpreted with an effect level of 0.25 and power level of 0.8, when significance was set at 0.05 [23]. Lastly, G*Power software confirmed that for a correlation, point biserial model, a sample size of 40 allowed data to be interpreted with an effect level of 0.4 when the power level was 0.8 and significance was set at 0.05 [23]. Subjects were recruited if they satisfied the following conditions: were college-aged (18–30 years); had a training history which included a resistance training age of at least one year (minimum of 3 h per week) [11,18,19,20], and were experienced with the movements of the BHT; were recreationally involved in a field or court sport (i.e., tennis, soccer, lacrosse, flag football, ultimate Frisbee, basketball, badminton, and volleyball), at least twice a week for the past two years; and were free from any lower-extremity injuries that could influence study participation [11]. No minimum relative strength for the BHT exercise was required for participation in the study. The university’s institutional review board (HSR-18-19-229) approved this study. The requirements of the study, inclusive of the risks and benefits associated with participation were provided to all subjects. Written informed consent was obtained from subjects prior to testing.

### 2.2. Procedures

Testing was conducted over three days with 48–72 h separating each session. Each subject completed the testing sessions at the same time of day, dependent upon their availability [11,22,24,25]. The first testing session initially required 1RM BHT testing and 505 COD speed test familiarization. The subsequent two testing sessions were completed in a counter-balanced order utilizing two different CAs (3 sets × 5 repetitions of BHT with 85% 1RM, and a CC of 6 min rest). Hence, half the sample performed the BHT first, and half performed the CC first, and then switched for the final testing session. Subjects were instructed to refrain from any intensive exercise or any form of stimulant and asked to maintain their typical dietary intake in the 24 h period prior to testing. 

During session one, the subject’s age, height, and body mass were taken. A stadiometer (Detecto, Webb City, MO, USA) was used to measure height, while electronic scales (Ohaus, Parsippany, NJ, USA) were used to measure the body mass of each subject. All subjects completed the same warm-up, which consisted of 5 min of jogging at a self-selected pace on a treadmill, followed by 10 min of dynamic stretching. Following this, subjects performed two trials of the 505 COD test to familiarize themselves for the next two sessions (all 505 COD speed trials were completed on the same indoor, wooden sprung basketball court). Once the 505 COD performances were completed, they performed the 1RM BHT test in an indoor weight room. During sessions 2 and 3, the subjects completed the same standardized dynamic warm-up as previously outlined, then at 5 and 2.5 min prior to the CA performed two trials of the 505 COD test (one per leg). The order of the trials was determined by the subject’s dominant leg (i.e., their preferred cutting leg) [26]. The testing order with regard to the turning leg was kept consistent for each subject across all trials. After baseline measurements, one of the two CAs (BHT or CC) was completed by the subject, before they completed the 505 COD test at 4, 8, 12, and 16 min post-CA [11,17,18,22,24,25,27,28,29]. Subjects wore their own athletic footwear for each testing session, and this same footwear was worn for all testing sessions.

### 2.3. Barbell Hip Thrust (BHT)

**Figure 1:**
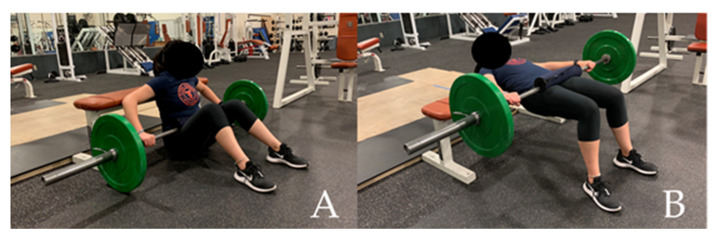
Starting (**A**) and vertical (**B**) position of the barbell hip thrust (BHT).

During session one, subjects performed a 1RM BHT to measure absolute and relative strength, and to determine the weight that was used for the 85% 1RM PAPE condition. To perform the BHT, subjects were instructed to start by sitting on the ground with their legs flat on the floor, feet shoulder-width apart, and their upper back against a padded exercise bench. A standard Olympic barbell and weight plates (Diamond Pro, Decatur, AL, USA), with a pad positioned on the bar for comfort [30], was placed above their lower legs, slightly below the knees. Once the subject positioned the barbell above their pelvis, they then assumed the start position of the exercise by bringing their heels toward the bench by flexing the knees (Figure 1A). Subjects then lifted their hips until their knee joint created a 90° angle with a vertical tibia (this was visually assessed by the researcher; Figure 1B). They held this position for one second before lowering the barbell in a controlled manner [30].

The procedures for finding the 1RM BHT were as follows. After the standard dynamic warm-up, the BHT warm-up sets completed by the subject consisted of 3 sets × 8 repetitions at 30%, 40%, and 50% of the subject’s perceived 1RM, with 2 min of rest provided between sets [17]. Once the warm-up sets were completed, a load equivalent to ~90–95% of the subject’s perceived 1RM was placed on the bar, and the subject complete a single repetition. After this, the weight was increased by approximately 5% and subjects completed single repetitions until the 1RM was attained, with 3 min rest provided between attempts [31,32]. In order for the repetition to be counted, the BHT had to be completed with good form (i.e., the knee joint had to reach a 90° angle with a vertical tibia), which was visually assessed by the researcher [17]. The 1RM was attained within 5 attempts. Verbal encouragement was provided to all subjects for each 1RM attempt. Absolute strength was taken as the 1RM load lifted; the 1RM was also scaled relative to body mass [31,32].

### 2.4. 505 Change-of-Direction Speed Test

**Figure 2:**
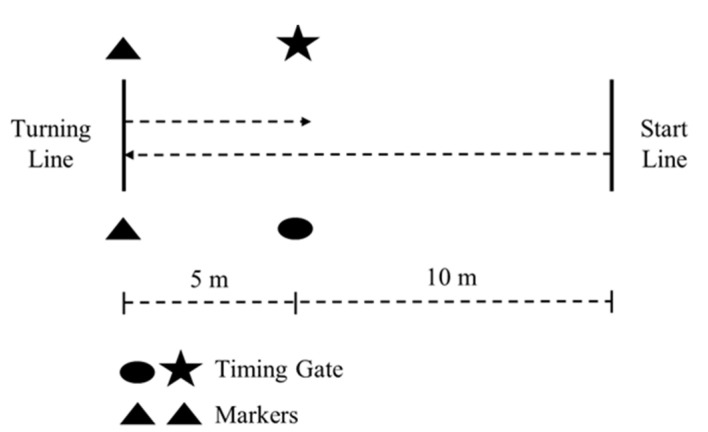
Structure of the 505 change-of-direction (COD) speed test.

The 505 COD speed test incorporates two 5 m sprints separated by a 180° turn (Figure 2). The protocol utilized within the current study is identical to that of previous research [11,26,33,34,35,36,37,38,39]. The subject began at the start line and accelerated through the timing gate (Brower Timing Systems, Draper, UT, USA) to the turning line, which was indicated by a line marked on the ground. The subject placed either their dominant or non-dominant foot, depending on the trial, on or behind the turning line, executed a 180° turn before sprinting back through the gate. If the subject failed to place part of their foot on or behind the turning line or turned off the incorrect foot, the trail was disregarded and reattempted after the required rest interval. The timing gates recorded the time to the nearest 0.01 second (s). The mean of the trials completed at each time point was used for analysis. At baseline however, the mean of the four trials (i.e., two trials at 5 min and two trials at 2.5 min pre-CA) was used for analysis [11,22,24,25,27,28,29]. At each time point, the dominant leg was tested first [11,26]. 

### 2.5. Post-Activation Performance Enhancement Interventions

As stated, following the dynamic warm-up, subjects performed two trials of the 505 COD speed test at 5 and 2.5 min prior to the CA with the mean of all CAs calculated to provide baseline data [11,22,24,25,27,28,29]. After recording the baseline data, subjects walked ~30 m to an indoor gym and performed one of two CAs. One session involved 3 sets × 5 repetitions of the BHT with 85% 1RM, with a 2 min rest between sets [17,18]. The concentric phase for each BHT repetition was to be performed as forcefully and rapidly as possible, while the bar was to be lowered with control in the eccentric phase [40]. For the CC, subjects were seated for 6 min, which was the approximate duration for the BHT CA [22,24,25]. After the CA, subjects walked ~30 m to the basketball court where the 505 COD speed test was performed and completed, with two trials at each of the following time points: 4, 8, 12, and 16 min post-CA [11,22,24,25,27,28,29]. The 4 min time was used as the start point as this was the initial time used in previous BHT PAPE research [17,18]. Subjects were not informed as to what their preceding 505 COD speed test times were to eliminate the influence of feedback [11,22]. The mean of the two trials performed at each time point was used for analysis. 

The following equation was utilized to calculate Post-CA COD performance percentage change: % Potentiation = Potentiated Variable (COD at 4, 8, 12, and 16 min) ÷ Unpotentiated Variable (average baseline) × 100. To interpret the Post-CA performance percentage change; a value less than 100 indicated a faster 505 COD speed performance (i.e., positive potentiation); a value greater than 100 indicated a slower 505 COD speed performance (i.e., negative potentiation); and a value equal to 100 indicated no change in 505 COD speed performance (i.e., no potentiation) [11,19,22,24,25].

### 2.6. Statistical Analysis

All analyses were determined via the Statistics Package for Social Sciences (Version 26.0; IBM Corporation, New York, NY, USA). Descriptive statistics (mean ± standard deviation (SD)) were calculated for all subjects. Q–Q plot analysis was utilized to determine the normality of the data [11,41,42]. Intraclass correlation coefficient (ICC) and coefficient of variation (CV) were determined to assess the reliability of the data. The criterion for reliability was set at ICC ≥ 0.70 and CV < 10% [43,44]. As both men and women can experience PAPE [12], the sexes were combined. This approach of combining the sexes with regard to PAPE has been utilized in previous research [10,11,25]. The statistical analysis performed was similar to that for Orjalo et al. [11]. To measure within-subject 505 COD speed performance across the assessed time points, a 2 × 5 repeated-measures ANOVA (condition (BHT and CC) × time (baseline, 4, 8, 12, and 16 min)) was used. A similar approach has been used in previous research [22,25,28,45]. To analyze the individual responses of each subject, regardless of the time point it occurred post-CA, best potentiated performance was analyzed [22,25,46]. A 2 (BHT and CC) × 2 (baseline and best 505) repeated-measures ANOVA was used for this analysis. If a significant *F* ratio was detected in any ANOVA calculations, post hoc pairwise tests were conducted using the Bonferroni adjustment procedure for multiple comparisons. The individual results of all participants were also described to allow for a more detailed examination of the effects of PAPE via the BHT upon COD speed, which may provide pertinent information to the strength and conditioning practitioner not possible via grouped analysis. 

To investigate relationships between absolute and relative strength measured by the 1RM BHT with percent 505 potentiation, partial correlations were used. Due to the established differences between women and men with regard to strength, power, and speed tests, partial correlations controlling for sex were utilized [37,47,48]. Similar to the clarifications stated by Orjalo et al. [11] and Dillman, Carpentier, and Stevens [49], sex was coded (males = 1; females = 2) to allow the partial correlation analysis to be conducted. The approach of utilizing partial correlations to control for the confounding effects of sex, so as to investigate the relationship between different fitness [11,37,50,51,52] and other variables [49,53,54] has been undertaken numerous times in previously published studies. The correlation strength was designated as follows: an *r* between 0 to ±0.3 was small; ±0.31 to ±0.49, moderate; ±0.5 to ±0.69, large; ±0.7 to ±0.89, very large; and ±0.9 to ±1, near perfect, for relationship prediction [55]. For all analyses, significance was set at *p* < 0.05.

## 3. Results

**Figure 3:**
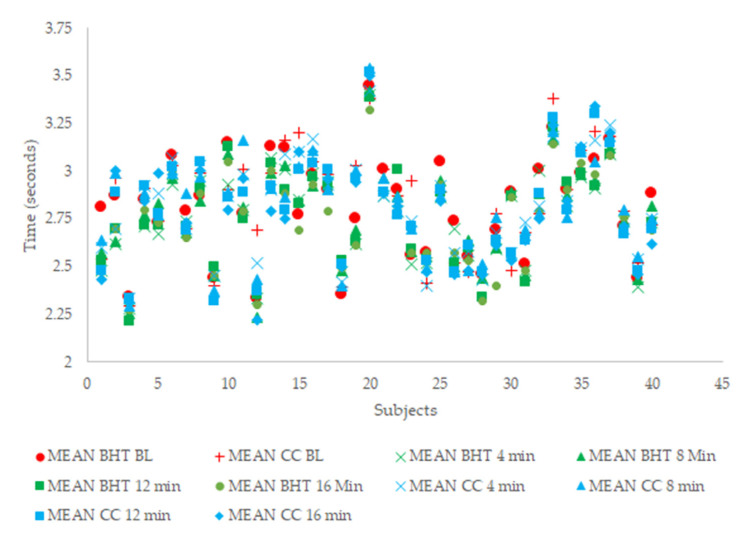
The mean, individual 505 change-of-direction speed test results across each condition and time-point. BHT = barbell hip thrust; CC = control condition; BL = baseline; min = minutes.

The Q–Q plot analysis determined that all assessed variables were normally distributed. All assessed variables demonstrated acceptable levels of reliability (ICC ≥ 0.70 and CV < 10%). Table 1 displays the descriptive data for the 505 COD speed test following each condition (i.e., BHT and CC). The percent potentiation is displayed in Table 2. When considering the 505 data recorded from 4 to 16 min post-CA relative to baseline, there was no significant main effect for condition (F_1,39_ = 3.402, *p* = 0.073, partial η^2^ = 0.183) or condition × time (F_4,36_ = 3.402, *p* = 0.170, partial η^2^ = 0.041). There was a significant main effect for time (F_4,36_ = 9.517, *p* < 0.001, partial η^2^ = 0.196). Post hoc analyses indicated that the pooled 505 data at 4 min (*p* = 0.005), 8 min (*p* = 0.014), 12 min (*p* = 0.005), and 16 min (*p* = 0.001) were all significantly faster than at baseline. Regarding the best potentiated 505 time, there was again no significant main effect for condition (F_1,39_ = 1.249, *p* = 0.271, partial η^2^ = 0.031) or condition × time (F_4,39_ = 1.125, *p* = 0.295, partial η^2^ = 0.028). There was a significant main effect for time (F_1,39_ = 67.651, *p* < 0.001, partial η^2^ = 0.634), with the best potentiated 505 time significantly (*p* < 0.001) faster than the baseline. The individual results of each subject across each condition and time point are presented in Figure 3. 

The mean 1RM BHT (absolute strength) for the subjects in this study was 156.26 ± 64.17 kg (males = 198.68 ± 55.87 kg; females = 113.83 ± 39.27 kg). Accordingly, the mean relative strength measure equaled 1.44 ± 0.24 kg·BM^−1^ (males = 2.50 ± 0.81 kg·BM^−1^; females = 1.76 ± 0.62 kg·BM^−1^). Table 3 displays the correlation data for absolute and relative strength and the percent potentiation for BHT and CC. There were no significant correlations between the lower-body strength measures and 505 percent potentiation for either of the CAs. 

## 4. Discussion

The purpose of this study was to determine whether 3 sets × 5 repetitions of BHT with 85% 1RM could potentiate COD performance as measured by the 505 COD speed test in college-aged men and women. Previous research has shown the benefit of this exercise and load for enhancing linear sprint performance [17,18]. The results provided some support to the hypothesis, as 505 COD speed test performance was acutely enhanced at every time point analyzed in this study. However, any acute improvements to the 505 COD test time that resulted from the use of the BHT as a CA were no different from a CC of 6 min rest. Further, and counter to other PAPE studies [12,17,18,20], the partial correlation data indicated no significant relationships between absolute and relative strength measured by the BHT and 505 percent potentiation. As will be discussed, these results were likely influenced by the training history of the subjects in this study who all had a minimum training age of one year. However, they were not high-level athletes with a training history consistent with elite sport. Nonetheless, the data presented could have practical application for strength and conditioning coaches. The BHT could be used as a CA with COD drills, as even though any enhancements were not different to a CC, a faster 505 COD test did result after the BHT. Consequently, this has implications for training efficiency in college-aged men and women, as strength and conditioning coaches can prescribe the BHT prior to COD drills, without a decrease in performance, while allowing training adaptations to still occur.

Previous research has shown that 3 sets × 5 repetitions of BHT with 85% 1RM can enhance sprinting speed over 15 m in male handball players [17] and over 20 m in male soccer players [18]. Interestingly, irrespective of the PAPE condition, 505 COD speed test times were faster at all time points relative to baseline. This also meant the best potentiated time following the BHT or CC was also faster than the baseline. These results may have occurred due to the training history of the subjects. The baseline 505 COD test times of the current subjects (BHT baseline = 2.82 ± 0.27 s; CC baseline = 2.84 ± 0.28 s) were relatively slower compared to those of higher-level athletes. For example, in elite rugby league players, Delaney et al. [33] found 505 COD test times of 2.21 ± 0.07 s and 2.23 ± 0.08 s for the dominant and non-dominant legs, respectively. Lockie et al. [39] found 505 COD test times of 2.24 ± 0.14 s and 2.20 ± 0.09 s for the left and right legs, respectively, in Division I collegiate men’s soccer players. Division I and II women’s soccer players had mean 505 COD test times of 2.40 ± 0.10 s and 2.60 ± 0.11 s, respectively [38]. The subjects from this study were clearly slower than elite and collegiate athletes, so potentially, they had a greater ceiling to improve with any form of stimulus. Although, it is important to note that differences in testing methodology between studies could explain part of the differences in 505 COD test times. Nonetheless, the current subjects may have been more sensitive to the BHT and even to the 6 min rest following a dynamic warm-up (i.e., the CC). This could have led to the general reduction in 505 COD test times post-CA for both analyzed conditions. Furthermore, it should also be noted that improvements in the 505 COD speed test following the BHT could be due to the placebo effect, whereby the act of the intervention led to performance enhancement [56]. However, given the results of this study and how they support previous research [17,18], it is likely the placebo effect was not an overriding factor.

What is notable is that even if 3 sets × 5 repetitions of BHT with 85% 1RM was not significantly different from the CC, it did lead to a faster 505 COD test performance at multiple time points. Especially in college-aged athletes, training time and exposure can be limited [57]. This puts a greater emphasis on training efficiency. The data from this study suggest that the BHT with a load focused on strength could be implemented prior to COD drills and may even led to a potentiation of COD speed. Given that a variety of training drills have been implemented in an attempt to improve COD speed [2,3,4], it is beneficial for strength and conditioning coaches to know that as long as recovery time is appropriate, heavy BHT could provide an acute positive impact on COD speed. Although, this improvement was similar to that with the CC within the current investigation.

Previous research has suggested that stronger individuals could experience greater PAPE [12,17,18,20]. However, results from this study are counter to these previous findings. There were no significant relationships between absolute and relative lower-body strength measured by the 1RM BHT with 505 COD test percent potentiation. In a similar sample of college-aged men and women, Orjalo et al. [11] found no relationships between vertical jump and lateral bound performance with 505 COD test PAPE following a CA of unweighted or weighted lateral bounds. Similar to Orjalo et al. [11], the training background of the subjects in this study may have influenced these results. The mean 1RM BHT for the college-aged subjects in this study was 156.26 ± 64.17 kg, and this compares favorably with that of collegiate male and female athletes (~150 kg) [58] and resistance-trained individuals (145.80 ± 33.51 kg) [59]. However, there were relatively high SDs for the 1RM BHT for both the men (±55.87 kg) and women (±39.27 kg) in this study. This would intimate that there was a spread of subjects with higher and lower strength levels in this study. Perhaps, future research should investigate the differences in COD speed percent potentiation and strength between “stronger” and “weaker” groups. Nonetheless, the subjects were also relatively slower in the 505 COD test, with examples provided in this discussion from elite [33] and collegiate [38,39] athletes. The strength of subjects in this study may not have effectively translated to their COD speed to the extent that there would be a superior ability to potentiate within the 505 COD test. This could also be complicated by the complexity of the COD action, despite both the BHT and COD requiring activation of the hip extensors. As even though the BHT could enhance the power output of the hip extensors due to the muscle’s contractile history [6], this may not lead to improved movement technique and strategies that are essential for a COD. Indeed, an effective direction change stresses multiple muscles in the lower body, which must generate force to produce effective biomechanics [60]. The combination of these factors could have limited the strength of relationships between absolute and relative strength and 505 COD test percent potentiation.

There are several limitations to this study that should be acknowledged. As noted, the subject population were recreational athletes. Higher-level athletes may respond differently to a CA [19], and the subjects in this study were slower in the 505 COD test compared to elite [33] and collegiate [38,39] athletes. Accordingly, the findings from this study may not apply to elite athletes. Only one strength exercise (BHT) with one load (85% 1RM) was analyzed in this study. Dello Iacono and Seitz [18] found that a BHT load that optimized peak power was potentially more beneficial for potentiating 20- m sprint performance in elite soccer players. It is possible that a BHT that optimized peak power could have potentiated the 505 COD test as well, and this requires investigation. Lastly, only one type of COD speed test was analyzed in this study. There are a variety of COD tests that incorporate different movement patterns [61]. Future research should detail whether the BHT could potentiate other COD speed tests featuring cuts made at different angles.

## 5. Conclusions

The current study revealed that 3 sets × 5 repetitions of BHT with 85% 1RM was able to enhance COD speed as measured by the 505 COD speed test in college-aged, recreationally trained men and women. This occurred at multiple time points from 4 to 16 min post-CA, but the changes in 505 time were not different from a those due to a CC of 6 min rest. Additionally, there were no significant relationships between absolute and relative lower-body strength measured by the 1RM BHT with 505 percent potentiation. Although the data may have been influenced by the training history of the participants, there are useful implications that can be drawn from this analysis of college-aged men and women. To improve training efficiency in college-aged men and women, strength exercises could be positioned before COD drills that feature actions such as those in the 505 COD test. COD speed could also be potentiated as long as the recovery time is appropriate.

## Figures and Tables

**Table 1:** Descriptive statistics (mean ± standard deviation (SD)) for the 505 change-of-direction speed test following the post-activation performance enhancement interventions (barbell hip thrust (BHT) and control condition (CC)).

Data Collection Time Points	BHT (s)	CC (s)
Baseline	2.82 ± 0.27	2.84 ± 0.28
4 min	2.76 ± 0.25	2.82 ± 0.26
8 min	2.77 ± 0.26	2.81 ± 0.27
12 min	2.77 ± 0.26	2.80 ± 0.28
16 min	2.74 ± 0.25	2.79 ± 0.30
Best	2.70 ± 0.25	2.74 ± 0.27

**Table 2:** Percent potentiation (%) compared to baseline across all time points post-barbell hip thrust (BHT) and control condition (CC).

Data Collection Time Points	BHT	CC
4 min	97.18 ± 4.04	99.47 ± 3.17
8 min	98.26 ± 3.02	99.30 ± 3.62
12 min	98.21 ± 3.48	98.76 ± 3.95
16 min	97.22 ± 3.46	98.27 ± 4.54
Best	95.97 ± 3.19	96.72 ± 3.82

**Table 3:** Correlation data between absolute (barbell hip thrust (BHT) one-repetition maximum (1RM) load) and relative (BHT 1RM load relative to body mass) strength and percent potentiation in the 505 following the post-activation performance enhancement conditions (BHT or control condition (CC)).

Condition	Absolute Strength		Relative Strength	
	*r*	*p*	*r*	*p*
BHT				
4 min	0.151	0.357	0.115	0.484
8 min	0.064	0.698	0.105	0.523
12 min	0.186	0.256	0.112	0.496
16 min	−0.027	0.872	0.009	0.956
Best	0.080	0.630	0.060	0.718
CC				
4 min	0.009	0.955	0.001	0.996
8 min	0.209	0.202	0.208	0.203
12 min	0.006	0.969	−0.011	0.945
16 min	0.020	0.906	0.044	0.790
Best	0.020	0.906	0.002	0.991

## Abbreviations

The following abbreviations are used in this manuscript:

COD	change-of-direction
PAPE	post-activation performance enhancement
CA	conditioning activity
RM	repetition maximum
m	meter
min	minute
h	hour
BHT	barbell hip thrust
CC	control condition
kg	kilograms
ANOVA	analysis of variance
kg·BM^−1^	kilogram per kilogram body mass
s	second
SD	standard deviation
*r*	correlation coefficient
*p*	significance
